# Fulvestrant is an effective and well-tolerated endocrine therapy for postmenopausal women with advanced breast cancer: results from clinical trials

**DOI:** 10.1038/sj.bjc.6601631

**Published:** 2004-03-05

**Authors:** I Vergote, J F R Robertson

**Affiliations:** 1 Department of Gynaecology and Gynaecological Oncology, University Hospitals, Herestraat 49, B-3000 Leuven, Belgium; 2 Department of Surgery, Nottingham City Hospital, Nottingham, UK

**Keywords:** fulvestrant, ‘Faslodex’, advanced breast cancer, anastrozole, tamoxifen

## Abstract

Fulvestrant (‘Faslodex’) is a new type of endocrine treatment – an oestrogen receptor (ER) antagonist that downregulates the ER and has no agonist effects. Early efficacy data from phase I/II trials have demonstrated fulvestrant to be effective and well tolerated. Two randomised phase III trials have compared the efficacy of fulvestrant and the aromatase inhibitor, anastrozole, in postmenopausal women with advanced breast cancer progressing on prior endocrine therapy. Fulvestrant (intramuscular injection 250 mg month^−1^) was found to be at least as effective as anastrozole (orally 1 mg day^−1^) for time to progression (5.5 *vs* 4.1 months, respectively (hazard ratio (HR): 0.95; 95.14% confidence interval (CI), 0.82–1.10; *P*=0.48)) and objective response 19.2 *vs* 16.5%, respectively; treatment difference 2.75%; 95.14% CI, −2.27 to 9.05%; *P*=0.31). More recently, fulvestrant has also been shown to be noninferior to anastrozole in terms of overall survival, with median time to death being 26.4 months in fulvestrant-treated patients and 24.2 months in those treated with anastrozole (HR: 0.97; 95% CI, 0.78–1.21; *P*=0.82). In a further randomised phase III trial, fulvestrant was compared with tamoxifen as first-line therapy for advanced disease in postmenopausal women. In the overall population, efficacy differences favoured tamoxifen and noninferiority of fulvestrant could not be ruled out. In the prospectively defined subset of patients with ER-positive and/or progesterone receptor-positive disease, there was no statistically significant difference between fulvestrant and tamoxifen. This paper reviews the efficacy and tolerability results from these trials.

Fulvestrant (‘Faslodex’) is a new type of endocrine treatment – an oestrogen receptor (ER) antagonist with no agonist effects (Wakeling *et al*, 1991). In trials designed to establish the biological activity of fulvestrant, conducted in postmenopausal patients with previously untreated breast cancer, fulvestrant produced significant reductions in cellular levels of the ER, the oestrogen-dependent progesterone receptor (PgR), and the tumour cell proliferation marker Ki67 (DeFriend *et al*, 1994; Robertson *et al*, 2001).

The favourable results obtained with fulvestrant in early trials led to the initiation of a phase III clinical trial programme conducted in postmenopausal women with hormone-sensitive advanced breast cancer. As second-line therapy, fulvestrant was compared with the third-generation aromatase inhibitor (AI) anastrozole. The results from these trials have led to the approval of fulvestrant for the treatment of postmenopausal women with hormone-sensitive advanced breast cancer following progression on prior endocrine therapy. As first-line hormonal therapy, fulvestrant has also been compared with tamoxifen.

## PHASE I/II STUDIES

### Clinical activity

Initial efficacy data for fulvestrant in 19 postmenopausal patients with tamoxifen-resistant advanced breast cancer demonstrated that fulvestrant produced a clinical benefit (CB, complete response+partial response+stable disease for a duration ⩾24 weeks) rate of 69% with a median duration of response (DoR) of 25 months (Howell *et al*, 1996a). In this study, CB was observed in six of the nine (67%) women who had progressed on prior tamoxifen therapy and in seven of the 10 (70%) women who had relapsed after treatment with tamoxifen as adjuvant therapy (Howell *et al*, 1996a). As predicted from preclinical data (Hu *et al*, 1993), these findings demonstrated that fulvestrant was not crossresistant with tamoxifen in the clinical setting.

### Tolerability

Fulvestrant was well tolerated with no serious drug-related adverse events (AEs) reported and no patients withdrawn due to toxicity. Local injection-site reactions were also uncommon (DeFriend *et al*, 1994; Howell *et al*, 1996a). In both studies, fulvestrant was associated with only minor systemic AEs, and the only AE reported by more than one patient was headache (six of 37 patients, 16.2%) (DeFriend *et al*, 1994). Fulvestrant had no effect on serum gonadotrophin, sex hormone-binding globulin levels (DeFriend *et al*, 1994; Howell *et al*, 1996a) or serum lipids (Howell *et al*, 1996a). Administration of fulvestrant was not associated with any alteration in the frequency of pre-existing night sweats or hot flushes, and none of these AEs were initiated during fulvestrant treatment (Howell *et al*, 1996a).

## PHASE III STUDIES: FULVESTRANT *VS* ANASTROZOLE

Two phase III trials (0020 and 0021) were conducted to compare the efficacy and tolerability of fulvestrant *vs* anastrozole in postmenopausal women with hormone-sensitive advanced breast cancer (Howell *et al*, 2002; Osborne *et al*, 2002). The majority of patients (⩾95%) had received prior treatment with tamoxifen, but a few had previously been treated with megestrol acetate, goserelin or a selective ER modulator (SERM; droloxifene, idoxifene or raloxifene).

Trial 0020 was a randomised, open-label trial conducted in Europe, South Africa and Australia. Trial 0021 was a double-blind, double-dummy study conducted in North America. The design of both trials was identical, except for a slight variation in the dosage delivery regimen, which was due to variations in clinical practice at that time between the US and the rest of the world. In trial 0020, fulvestrant 250 mg was administered as a 1 × 5 ml intramuscular (i.m.) injection and in trial 0021 as 2 × 2.5 ml i.m. injections.

### Efficacy

**Figure 1:**
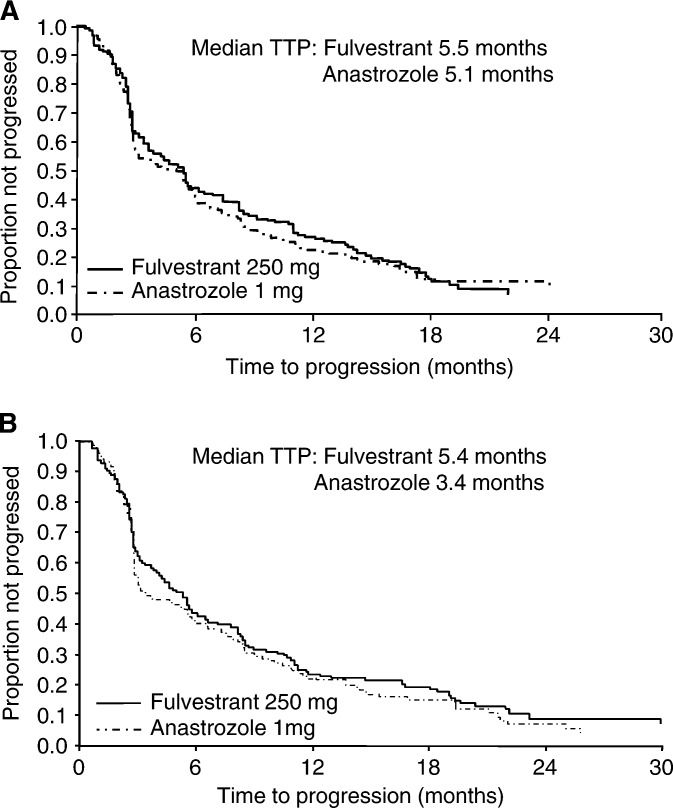
Kaplan–Meier estimates of TTP for fulvestrant *vs* anastrozole: (**A**) trial 0020, (**B**) trial 21 (Howell *et al*, 2002; Osborne *et al*, 2002). Reprinted with permission from the American Society of Clinical Oncology.

**Figure 2:**
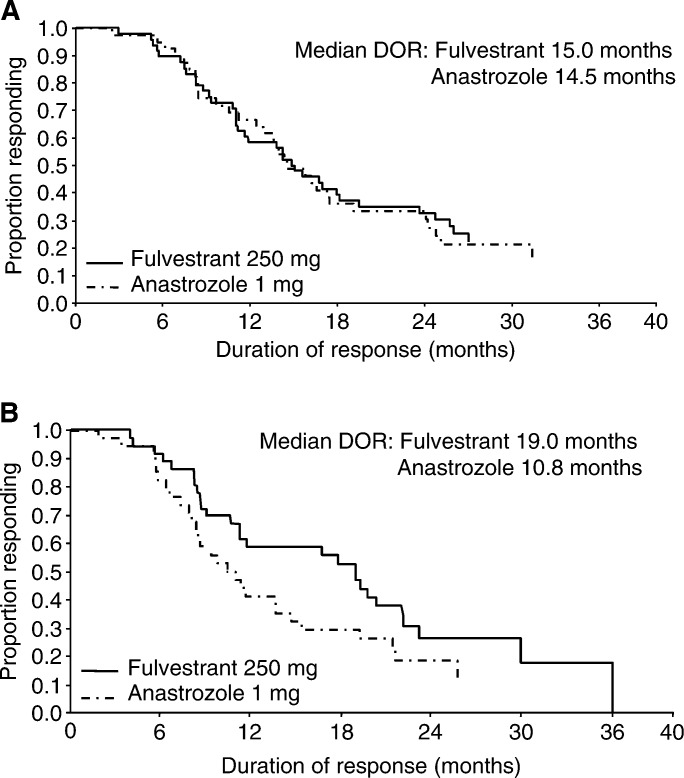
Kaplan–Meier estimates of DoR for fulvestrant *vs* anastrozole (responding patients only): (**A**) trial 0020, (**B**) trial 0021 (Howell *et al*, 2002; Osborne *et al*, 2002). Reprinted with permission from the American Society of Clinical Oncology.

In trial 0020, after a median follow-up of 14.4 months, the median time to progression (TTP) was 5.5 months for fulvestrant and 5.1 months for anastrozole (HR: 0.98; 95.14% CI, 0.80–1.21; *P*=0.84) (Figure 1A

). The objective response (OR) rate was 20.7% for fulvestrant and 15.7% for anastrozole (odds ratio: 1.38; 95.14% CI, 0.84–2.29; *P*=0.20). To obtain more complete information on DoR, further follow-up was performed. At a median follow-up of 22.6 months, the median DoR (from randomisation to progression in responding patients) was 15.0 months for fulvestrant and 14.5 months for anastrozole (Figure 2A

) (Howell *et al*, 2002). A survival analysis from this study showed that fulvestrant was noninferior to anastrozole with respect to overall survival, with the median time to death being 26.4 months in fulvestrant-treated patients and 24.2 months in those treated with anastrozole (HR: 0.97; 95% CI, 0.78–1.21; *P*=0.82) (Howell *et al*, 2003).

In trial 0021, patients were followed up for a median of 16.8 months. The median TTP was 5.4 months for fulvestrant and 3.4 months for anastrozole (HR: 0.92; 95.14% CI, 0.74–1.14; *P*=0.43) (Figure 1B). OR was 17.5% for both fulvestrant and anastrozole (odds ratio: 1.01; 95.14% CI, 0.59–1.73; *P*=0.96). After an extended median follow-up of 21.3 months, the median DoR was 19.0 months for fulvestrant and 10.8 months for anastrozole (Figure 2B) (Osborne *et al*, 2002).

Both trials were prospectively designed to allow combination of results. After a median follow-up of 15.1 months, combined data from 851 patients showed a median TTP of 5.5 and 4.1 months (HR: 0.95; 95.14% CI, 0.82–1.10; *P*=0.48) and OR rates of 19.2 and 16.5% for fulvestrant and anastrozole, respectively (difference in response rates: 2.75%; 95.14% CI −2.27 to 9.05%; *P*=0.31) (Robertson *et al*, 2003). Analysis of DoR from an extended median follow-up of 22.1 months showed the median DoR to be 16.7 months for fulvestrant and 13.7 months for anastrozole (Robertson *et al*, 2003). In a further analysis of DoR (mean DoR) that included all randomised patients (defined for responders as the onset of response to disease progression, and for nonresponders as zero), DoR was 30% greater for fulvestrant compared with anastrozole (ratio of average response durations: 1.30; 95% CI, 1.13–1.50; *P*<0.01) (Robertson *et al*, 2003).

Of the 851 patients in the combined analyses of both trials, visceral metastases were present in 381 (44.8%) patients. After a median follow-up of 15.1 months, OR and CB were similar between fulvestrant and anastrozole in patients with visceral metastases (OR: 15.7 *vs* 13.2%; CB: 38.2 *vs* 37.4% for fulvestrant *vs* anastrozole, respectively) and in those with no visceral metastases (OR: 21.9 *vs* 19.3%; CB: 47.6 *vs* 43.8%) (Mauriac *et al*, 2003). A further retrospective analysis of the combined data from these trials evaluated response to subsequent therapy in patients gaining CB or no CB (stable disease <24 weeks+disease progression) from fulvestrant treatment. Of the 54 patients deriving CB from fulvestrant, who also received further endocrine therapy, 25 patients derived CB from subsequent treatment. Similarly, 18 of the 51 patients who did not derive CB from fulvestrant went on to gain CB from subsequent endocrine treatment, demonstrating that patients may retain sensitivity to other endocrine agents after progression on fulvestrant (Vergote *et al*, 2003).

### Tolerability

In both trials, fulvestrant was well tolerated. At the outset of the trials, seven AEs were considered relevant to endocrine therapy and were predefined for statistical analysis. These were: gastrointestinal disturbances, hot flushes, vaginitis, weight gain, thromboembolic disease, urinary tract infection and joint disorders (including arthralgia, arthrosis and arthritis). In the combined analysis, the incidences of these predefined AEs were similar for fulvestrant and anastrozole, except joint disorders, the incidence of which was significantly lower for fulvestrant compared with anastrozole (5.4 *vs* 10.6%; *P*=0.0036) (Table 1

**Table 1:** Incidences of predefined adverse events for fulvestrant *vs* anastrozole (combined analysis) (Robertson *et al*, 2003)

	**Fulvestrant (*n*=423)**		**Anastrozole (*n*=423)**		
	***n***	**%**	***n***	**%**	***P-*value**
Gastrointestinal disturbances	196	46.3	185	43.7	0.53
Hot flushes	89	21.0	87	20.6	0.91
Joint disorders	23	5.4	45	10.6	0.0036
Thromboembolic disease	15	3.5	17	4.0	0.68
Urinary tract infection	31	7.3	18	4.3	0.06
Vaginitis	11	2.6	8	1.9	0.51
Weight gain	4	0.9	7	1.7	0.35

Copyright © (2003) American Cancer Society. Reprinted with the permission of Wiley-Liss, Inc., a subsidiary of John Wiley & Sons, Inc.

). The most common AEs in both treatment groups, irrespective of the relationship to study medication, were nausea (26.0 *vs* 25.3% for fulvestrant and anastrozole, respectively), asthenia (22.7 *vs* 27.0%), pain (18.9 *vs* 20.3%), vasodilatation (17.7 *vs* 17.3%) and headache (15.4 *vs* 16.8%). Withdrawals due to drug-related AEs were reported in 0.9% of patients treated with fulvestrant and in 1.2% of those treated with anastrozole (Robertson *et al*, 2003).

### Quality of life

The maintenance of quality of life (QoL) is an important consideration when making treatment choices for patients with advanced breast cancer. In both phase III trials, the QoL was assessed using the Functional Assessment of Cancer Therapy – Breast (FACT-B) questionnaire. This tool is a sensitive measure for evaluating physical, functional, social and emotional well-being and comprises the FACT-G ‘general’ QoL tool for cancer patients plus the Breast Cancer Subscale (Cella *et al*, 1993; Brady *et al*, 1997). The treatment outcome index (TOI), within the FACT-B questionnaire, is the sum of the well-being and breast cancer subscales. In both trials, QoL was maintained over time with no statistically significant difference between anastrozole and fulvestrant (Howell *et al*, 2002; Osborne *et al*, 2002).

## PHASE III STUDY: FULVESTRANT *VS* TAMOXIFEN

Fulvestrant 250 mg (delivered as a once-monthly 5 ml i.m. injection) has been compared with tamoxifen 20 mg (orally, once daily) in a trial conducted in postmenopausal women with ER-positive and/or PgR-positive or ER/PgR-unknown advanced breast cancer who had not been treated with prior endocrine therapy or chemotherapy for advanced disease. This study was a double-blind, randomised, parallel-group, double-dummy trial conducted at 171 centres in 26 countries throughout the world, including Europe, North and South America, Africa and Australia.

Of the 587 patients who were randomised to treatment, 78% of patients in the fulvestrant group and 75.2% of those in the tamoxifen group had received no prior tamoxifen in the adjuvant setting.

### Efficacy

In the overall population, after a median follow-up of 14.5 months, there was no significant difference between the two treatments for TTP (median TTP: 6.8 *vs* 8.3 months for fulvestrant and tamoxifen, respectively; HR: 1.18; 95% CI, 0.98–1.44; *P*=0.09). However, observed differences in other efficacy end points favoured tamoxifen and noninferiority of fulvestrant could not be demonstrated (Robertson *et al*, 2002).

In a prospectively planned analysis of patients with ER-positive and/or PgR-positive tumours (78.9% of fulvestrant-treated patients and 77.4% of tamoxifen-treated patients), in the population intended for treatment with endocrine therapy, the TTP was similar between the two treatments (median TTP: 8.2 *vs* 8.3 months for fulvestrant and tamoxifen, respectively; HR: 1.10; 95% CI, 0.89–1.36; *P*=0.39). The OR rate in this hormone receptor-positive subgroup was 33.2% for fulvestrant and 31.1% for tamoxifen (Robertson *et al*, 2002).

Previous studies have suggested that patients with ER-positive and PgR-positive breast tumours have a more active ER and have demonstrated that these patients are more likely to benefit from endocrine therapy compared with patients with ER-positive/PgR-negative tumours (Wittliff, 1984; Jakesz *et al*, 2002; Bardou *et al*, 2003). In an exploratory analysis of only those patients with ER-positive and PgR-positive tumours (approximately 42% of the trial population), the median TTP was 11.4 months for fulvestrant and 8.5 months for tamoxifen (HR: 0.85; 95% CI, 0.63–1.15; *P*=0.31) and OR was significantly higher for fulvestrant compared with tamoxifen (44.3 *vs* 29.8%; *P*=0.02). Furthermore, 20 out of 35 patients who responded to first-line treatment with fulvestrant and provided follow-up information on subsequent treatments retained sensitivity to subsequent therapies including anastrozole, letrozole, fadrozole, tamoxifen and megestrol acetate (Howell and Robertson, 2002).

### Tolerability

Both treatments were well tolerated. The incidence of prospectively defined AEs of gastrointestinal disturbances (nausea, vomiting, diarrhoea and constipation), vaginitis and thromboembolic disease was similar between fulvestrant and tamoxifen. However, the incidence of hot flushes was lower in patients treated with fulvestrant compared with those treated with tamoxifen (*P*=0.0501).

## DISCUSSION

Two pivotal phase III trials have demonstrated that fulvestrant is well tolerated and is at least as effective as the third-generation AI anastrozole in patients with advanced breast cancer who have progressed on prior endocrine therapy. A prospectively planned combined analysis from these trials demonstrated that fulvestrant is at least as effective as anastrozole with respect to TTP, OR and DoR. Although investigations of the 1 × 5 ml and the 2 × 2.5 ml injections have demonstrated that the pharmacokinetic profiles of these doses are comparable (Robertson and Harrison, 2003), recent adjustments in US clinical practice have meant that fulvestrant is now generally administered as a 1 × 5 ml injection. This single parenteral mode of administration may offer potential benefits in terms of patient compliance (particularly for elderly patients) compared with the oral dosing regimens of most other endocrine therapies.

As first-line treatment of advanced breast cancer in postmenopausal women with ER-positive and/or PgR-positive tumours, fulvestrant appears to have a similar efficacy to tamoxifen. In patients with ER-positive and PgR-positive tumours, a subset likely to be sensitive to the positive benefits of endocrine treatments, a retrospectively derived analysis of the data was suggestive of a possible benefit for fulvestrant compared with tamoxifen in terms of OR. However, these latter findings require further investigation so that the patient population most likely to benefit from fulvestrant in the first-line setting can be established.

Fulvestrant is the only ER antagonist that has demonstrated unequivocal efficacy in patients with tamoxifen-resistant advanced breast cancer. Although other antioestrogens, that is, some of the SERM-like compounds, have shown limited efficacy in the first-line treatment of advanced breast cancer, they are relatively ineffective in patients who are resistant to tamoxifen (Stenbygaard *et al*, 1993; Pyrhonen *et al*, 1994; Howell *et al*, 1996b; Buzdar and Hortobagyi, 1998). This provides evidence that fulvestrant has a novel mode of action and illustrates that it is an appropriate therapeutic choice for patients with hormone-sensitive advanced breast cancer who have received prior treatment with endocrine therapy (including tamoxifen).

In summary, as second-line therapy for postmenopausal women with ER-positive and/or PgR-positive advanced breast cancer who have progressed on prior endocrine treatment, fulvestrant is clearly an effective therapy that offers equal efficacy to anastrozole.
